# Potential roles for microbial endophytes in herbicide tolerance in plants

**DOI:** 10.1002/ps.4147

**Published:** 2015-10-09

**Authors:** Catherine Tétard‐Jones, Robert Edwards

**Affiliations:** ^1^ School of Agriculture, Food and Rural DevelopmentNewcastle UniversityNewcastleUK

**Keywords:** herbicide selectivity, xenobiotics, allelochemicals, microbial symbiosis, defence priming, multiple herbicide resistance

## Abstract

Herbicide tolerance in crops and weeds is considered to be monotrophic, i.e. determined by the relative susceptibility of the physiological process targeted and the plant's ability to metabolise and detoxify the agrochemical. A growing body of evidence now suggests that endophytes, microbes that inhabit plant tissues and provide a range of growth, health and defence enhancements, can contribute to other types of abiotic and biotic stress tolerance. The current evidence for herbicide tolerance being bitrophic, with both free‐living and plant‐associated endophytes contributing to tolerance in the host plant, has been reviewed. We propose that endophytes can directly contribute to herbicide detoxification through their ability to metabolise xenobiotics. In addition, we explore the paradigm that microbes can ‘prime’ resistance mechanisms in plants such that they enhance herbicide tolerance by inducing the host's stress responses to withstand the downstream toxicity caused by herbicides. This latter mechanism has the potential to contribute to the growth of non‐target‐site‐based herbicide resistance in weeds. Microbial endophytes already contribute to herbicide detoxification in planta, and there is now significant scope to extend these interactions using synthetic biology approaches to engineer new chemical tolerance traits into crops via microbial engineering. © 2015 The Authors. *Pest Management Science* published by John Wiley & Sons Ltd on behalf of Society of Chemical Industry.

## 1 INTRODUCTION

**Figure 1:**
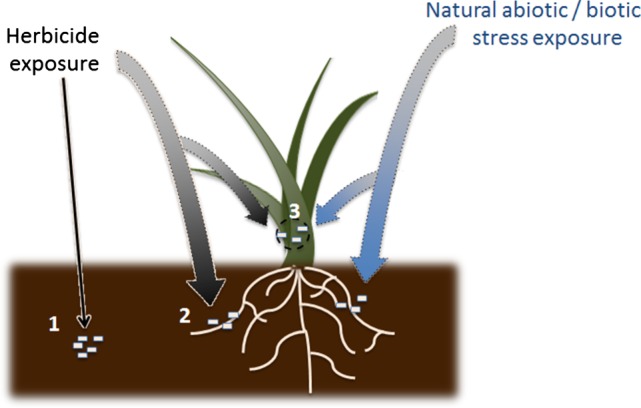
Mechanisms that microbes can contribute to reduction in herbicide efficacy by metabolism, either by (1) free living in soil, (2) cohabiting and cometabolising within plant tissues or (3) induction of stress responses in the host plant to withstand the downstream toxicity caused by herbicides, thereby making them more tolerant.

The relative tolerance of crops and weeds to herbicides is commonly associated with either fundamental differences in the biochemical processes targeted or the relative rates of herbicide detoxification.^1^, ^2^ Traditionally it is considered that herbicide selectivity is defined by plant physiology and biochemistry alone. However, microbes living in both symbiotic and pathogenic relationships with host plants are well known to enhance their resistance to further infection as well as increase tolerance to environmental stresses.^3^, ^4^ This microbial ‘priming’ of plant stress resistance is most commonly associated with natural abiotic (nutrient deficiency, drought, extreme temperature) and biotic (infection) stress, with effects on herbicide tolerance receiving little attention. As herbicide injury is related to specific subsets of biotic and abiotic stress, we propose that endophytes or rhizobacteria present in plant tissues or free‐living microbes living in intimate proximity can induce latent stress signalling pathways that enhance endogenous ‘resistance’ to chemical stress.^3^, ^5^ It is also possible that such interactions will reduce chemical abiotic stress tolerance, although that possibility falls outside the scope of this short review. Instead, we address the hypothesis that plant–microbe symbiosis results in the ‘priming’ of cellular defences that make the host more resistant to the downstream toxicity caused by herbicides (Fig. 1). This may be particularly relevant in cases where plants coexist with microbes that produce phytotoxic secondary metabolites – some of which have been developed into or used as a template for synthetic herbicidal compounds with new modes of action.^6^ Furthermore, bacteria found in the environment have a well described ability to metabolise and detoxify xenobiotics.^7^, ^8^ In fact, the microbial degradation of xenobiotics, including pesticides, polycyclic aromatic hydrocarbons and fuels, is widely utilised for the bioremediation of contaminated land and water systems.^7^, ^8^ As a logical extension to these reports, if xenobiotic‐detoxifying bacteria were present in plants as endophytes, they would be potentially able to contribute to herbicide detoxification and hence selectivity (Fig. 1).

In this article, we have reviewed currently documented studies of bitrophic relationships between plants and microbes contributing to relative herbicide tolerance. Here we define bitrophic as an intimate plant–microbe interaction between bacteria/fungi that are present either within or on the surface of plants, or in the rhizosphere. Such microbial innoculants could either be derived from free‐living colonies or generationally transmitted through association with seeds. Where known, we have explored the mechanisms underpinning these chemical tolerance symbioses and their evolutionary origin. Three scenarios have been envisaged whereby plant–microbe interactions can contribute to herbicide tolerance: (1) metabolism by free‐living soil microbes, reducing the availability of pre‐emergence herbicides; (2) cometabolism by plant‐associated endophytes; (3) induction of plant stress responses by endophytes, leading to enhanced downstream resistance to chemical injury (Fig. 1). Finally, we have examined the potential for endophytes to contribute to the adaptation of herbicide resistance in weeds, as elicited by one or more of these mechanisms.

## 2 HERBICIDE METABOLISM BY FREE‐LIVING SOIL MICROBES

The degradation of xenobiotics by microbes is an evolutionary adaptation that utilises their metabolic diversity either to enable their survival in the presence of toxins or to utilise them as a source of carbon, phosphorus, sulphur or nitrogen.^9^ This ability has been exploited in the bioremediation industry to assist in the reclamation of brown‐field sites through the microbial decontamination of persistent organic pollutants, including those arising from the manufacture of herbicides.^9^ In addition to applications in bioremediation, the microbial detoxification of xenobiotics in the soil is a key factor determining the environmental fate and persistence of registered agrochemicals, including herbicides, and as such is of interest to both the chemical companies and regulatory bodies.^10^, ^11^ In the case of herbicide metabolism, a diverse range of microbial biotransformations of herbicides have been described, notably linked to oxidation, reduction, bond cleavage by lyases and hydrolytic reactions (Table 1). The ability of microbes to degrade herbicides and other pesticides appears to derive from the ability to detoxify natural products that in some way resemble xenobiotics. For example, the diversity of rhizobacterial *tfdA*‐like genes linked to 2,4‐D metabolism appears to have evolved to detoxify natural analogues of the herbicide long before the first use of the compound in the early 1940s.^12^ In several cases, multiple degradation pathways have evolved to compete effectively to metabolise the agrochemical. For example, organophosphorus compounds such as glyphosate (Table 1), accounting for a third of worldwide pesticide sales, can be degraded by multiple documented pathways by microbes in the rhizosphere.^9^ Similarly, the thiocarbamate herbicide molinate is degraded by multiple pathways in both bacterial and fungal species, depending on environmental conditions.^13^ Under aerobic conditions, the majority of molinate is degraded by oxidative routes in the presence of an additional cometabolic carbon source. However, under anaerobic conditions, complete mineralisation of molinate as a single carbon and energy source has been attributed to a hydrolytic mechanism.^13^

**Table 1:** Molecular pathways leading to microbial herbicide metabolism/cometabolism

Herbicide	Genes/pathway	Reference
	*Metabolism by free‐living microbes*	
2,4‐D (glycolate)	*tfdA*‐like genes (*α*‐ketoglutonate‐dependent dioxygenase pathway), producing 2‐chloromaleylacetate	62
Atrazine (triazine)	Oxidative–hydrolytic and hydrolytic pathways, producing cyanuric acid	63
Glyphosate (organophosphorus)	Several microbial pathways documented:	20, 27, 64, 65
	enzyme EPSPS (5‐enol‐pyruvylshikimate‐3‐phosphate synthase) from *Agrobacterium* sp. strain CP4‐associated target‐site resistance degradation by the C–P lyase pathway/phosphonatase pathway (phosphonate‐degrading enzymes), producing aminomethyl phosphonic acid amino‐methyl phosphonic acid pathway by *Penicillium notatum* glyphosate *N*‐acetyltransferase (GAT) acetylation, producing *N*‐acetylglyphosate (NAG)	
Molinate (thiocarbamate)	Several pathways by diverse bacterial and fungal isolates, either:	13
	*oxidation*: (a) of the ethyl moiety of molinate, with the formation of molinate alcohol and molinate acid: (b) of the azepane ring, with the formation of hydroxyl‐ and oxo‐molinate derivatives; (c) of the sulphur atom and subsequent cleavage of the C–S bond, with the formation of hexamethyleneimine and an *S*‐ethyl derivative *hydrolysis*: cleavage of the thioester bond of molinate by molinate hydrolase (MolA), producing ethanethiol and azepane‐1‐carboxilate (ACA)	
Sulfonylureas	Hydrolysis of the sulfonylurea linkage (pH dependent) via either acid‐catalysed cleavage or base‐catalysed contraction/rearrangement. Produces CO_2_ + corresponding aryl sulphonamide and aminoheterocyclic portions	66
	*Cometabolism by plant‐associated microbes*	
Alachlor (chloroacetanilide)	Glutathione *S*‐transferase (GST)‐mediated metabolism	67
2,4‐D, atrazine, aminotrizole, pentanochlor (anilide)	BphK^LB400^‐mediated metabolism (dechlorination). Note: similarity of BphK^LB400^ protein sequence to GST	33

While removal of chemical residues from the environment has many positive features, the microbial degradation of pre‐emergence herbicides in the soil can ultimately regulate their activity in weed control applications. For example, when applied to the soil, 2,4‐D is known to have a short half‐life on account of complete microbial degradation.^12^ Rates of degradation are dependent on agronomic practice and environmental conditions, being accelerated by the presence of organic matter and by increases in temperature.^11^ Accelerated metabolism is also seen in soils repeatedly treated with the same herbicide chemistry, caused by selection for degrading microbial populations.^14^, ^15^, ^16^ In spite of the obvious potential for the rhizosphere to degrade pre‐emergence herbicides, there are few reports of how this environmental detoxification determines their efficacy in the field. This presumably reflects the relatively short periods of time between application and weed control. However, with the increasing reliance on pre‐emergence chemistries to help counteract the build‐up of resistance to post‐emergence herbicides in weeds of cereal crops, this would be an interesting area for future research.^17^

A particular area of interest in free‐living microbes and herbicide detoxification is their potential as a source of useful genes both to produce herbicidal compounds and to confer tolerance to them in genetically transformed plants (similar to the discovery of antimicrobial compounds).^6^ In a classic example, two bacterial genes that play a role in both biosynthesis of and self‐defence against bialaphos and its metabolite phosphinothricin (common herbicide name glufosinate) in Liberty Link™ GM crops were originally isolated from soil bacteria *Streptomyces hygroscopicus* (bialaphos resistance, *bar* gene) and *S. viridochromogenes* (phosphinothricin acetyltransferase, *pat* gene).^6^, ^18^, ^19^ More recently, crop tolerance to multiple herbicides has been achieved by pyramiding bacterial detoxification genes, notably through the use of GAT/HRA crop technology.^20^ This combines a glyphosate acetyltransferase (*gat*) gene derived from a naturally occurring soil bacterium (*Bacillus licheniformis*) along with a highly resistant acetolactate synthase (HRA) that shows insensitivity to all classes of herbicides active against this target.^20^

## 3 HERBICIDE COMETABOLISM BY PLANT‐ASSOCIATED MICROBES

Of immediate relevance to tolerance is the potential role of plant‐associated microbes (endophytes and rhizobacteria) in herbicide cometabolism within the tissues of crops and weed species. A wide range of endophytic and root‐surface‐colonising bacteria (rhizobacteria), notably drawn from *α*, *β* and *γ* subdivisions of the class Proteobacteria, have been shown to protect plants from herbicides by contributing to their metabolism.^16^, ^21^, ^22^, ^23^, ^24^, ^25^ Several studies have concentrated on the endophyte and rhizobacterial strain *Pseudomonas putida* POPHV6, originally isolated from the stem tissue of poplar.^21^ Rhizobial inoculation of pea plants with POPHV6 enhanced the disappearance of 2,4‐D from soil and reduced the translocation of the herbicide into aerial tissues.^26^ The POPHV6 strain was found within both the root and aerial tissues, suggesting that it was contributing to the metabolism of the herbicide throughout the plants. Other known rhizobacteria isolated from 2,4‐D‐treated soils and capable of degrading the herbicide in plant inoculation experiments included *Burkholderia cepacia*, *Cupriavidus pinatubonensis* JMP134 and *Ralstonia eutropha* JMP134.^12^, ^21^, ^22^ Similarly, rhizobacteria and endophytic bacteria are also known to detoxify glyphosate and the *s*‐triazine atrazine with soil application, favouring the enrichment with herbicide degrading species.^23^, ^27^, ^28^

In addition to conventional crops, attention has also focused on the effects of herbicides on rhizobial communities in GM crops engineered to be herbicide resistant, which are subject to repeated usage by otherwise non‐selective chemistries. Thus, metagenomics has been used to monitor microbial diversity in maize lines genetically modified to be tolerant to glyphosate.^29^ Glyphosate application altered the rhizobial population profile, with recovery to the pretreatment state strongly dependent on the soil type. In a further study, the effect of a range of herbicide treatments on the rhizobial communities associated with maize transformed to be both glyphosate tolerant and insect resistant, the latter through the expression of the *Bt* gene, was determined by sequencing the 16S rRNA genes present.^30^ These results showed that some combinations of selective herbicides, such as mesotrione and metalochlor, when coapplied with glyphosate, showed little effect on the microbial communities, whereas other mixtures containing acetochlor/terbuthylazine and aclonifen/isoxafutole were disruptive to the rhizobacterial populations over the long term.^30^ While these studies do not shed light on the potential involvement of endophytes in contributing to herbicide detoxification in the crop, they add interesting insights into the potential effects on symbiotic relationships between soil‐living bacteria, plants and herbicide tolerance in populations increasingly exposed to (i) repeated use of a single compound based on the use of GM‐derived resistance technology^29^, ^30^ and (ii) the use of formulations applied to exploit new selectivity profiles and combat resistance.^17^

With the available evidence demonstrating that endophytes are indeed able to contribute to xenobiotic detoxification in plants, this poses the question as to whether this can be usefully exploited to engineer pesticide degradation in crops. Such an approach may have merits in the case of consumer perceptions of food safety. Thus, endophytes provide a means indirectly to modify crop health and growth traits, thus circumnavigating current public acceptance issues associated with eating stably GM‐transformed plant products.^31^ To date, this microbial route to engineering chemical tolerance technology has been limited to introducing novel phytoremediation traits into crops. For example, the introduction of the plasmid (pTOM) encoding for proteins involved in toluene degradation from one strain of *Burkholderia cepacia* G4 into a related natural endophyte of yellow lupine (*B. cepacia* L.S. 2.4) resulted in enhanced degradation of the solvent in plant trials.^32^ In the case of herbicides, endophytic bacteria producing the protein BphKLB400 (transferred from *Burkholderia xenovorans* LB400) endow pea plant hosts with the ability to degrade several pesticides (Table 1). BphKLB400 shows sequence similarity to glutathione transferases (GSTs), which are well known for their involvement in xenobiotic detoxification and in the case of the bacterial enzymes are able to catalyse dechlorination reactions.^33^ Mutation of an amino acid within the putative active site of BphKLB400 increased the GST activity of bacterial cell extracts towards a number of chlorinated organic substrates, including commonly used pesticides, suggesting that such detoxification traits could be evolutionarily selected for via exposure to repeated herbicide applications.^33^

## 4 INDUCTION OF PLANT STRESS RESPONSES BY ASSOCIATED MICROBES

It is now well established that rhizobacteria and endophytic microbes can induce the host plant to possess enhanced resistance to pests (insects, root nematodes), pathogens and abiotic stresses (e.g. drought, salinity, heat, nutrient limitation).^4^, ^34^, ^35^, ^36^, ^37^ Known mechanisms of this induced resistance in the host include the rhizobacterial priming of existing plant defence responses to pathogens and insects, termed induced systemic resistance (ISR), and the production of alkaloids in fungal endophytes that are toxic to invertebrate pests.^37^, ^38^ While most of the available literature has pointed towards bitrophic interactions between microbes and plants influencing biotic and abiotic stress, there is also tantalising evidence that these interactions can be influenced at the tritrophic level. As a notable example, the prevalence of viruses in fungi (mycoviruses) and their influence on mutualistic relationships with plants has attracted recent attention.^39^, ^40^ The ability of the grass *Dichanthelium lanuginosum* to survive soil temperatures ranging between 38 and 65 °C in Yellowstone National Park was directly linked to an association with the fungus *Curvularia protuberata* and its mycovirus *Curvularia* thermal tolerance virus (CThTV).^40^ Although heat tolerance in *D. lanuginosum* and other hosts, including tomato, was shown to be dependent on mycoviral infection of the fungal endophyte, the specific role of the CThTV and the mechanism by which it induced plant thermotolerance have not been elucidated. To explore the virus–fungus aspect of the interaction, transcriptomics was utilised to compare CThTV‐infected and non‐infected strains of *C. protuberata*.^41^ In culture, the virus‐infected fungus was able to survive at up to 38 °C, with differential gene expression putatively associated with increased fungal fitness. Candidate virus‐induced fungal heat stress genes included those encoding scytalone dehydratase (melanin biosynthetic pathway), heat shock proteins, glutathione transferases and enzymes involved in the biosynthesis of known osmoprotectants (trehalose, glycine betaine, taurine).^41^ While these results indicate that viruses can induce the expression of fungal genes that function in heat resistance, speculation remains over how this translates to the original finding of extreme tritrophic thermotolerance at up to 65 °C. Potentially, plants provide a protective habitat from direct heat (dehydration) exposure required for tolerance to higher temperatures, while the fungus provides virus‐induced heat tolerance proteins and metabolites.

Exploring the paradigm that endophytes can induce complex innate stress tolerance mechanisms in plants poses the intriguing question as to whether such plant–microbe interactions can influence tolerance to herbicides. This is particularly relevant given the recent proposals that safeners, compounds that enhance herbicide tolerance in cereals, use signalling pathways, including those involving jasmonic acid, oxylipins and salicylic acid, previously described as regulating wounding and systemic resistance responses.^42^ While these claims are yet to be substantiated, there is clear evidence that safener responsive genes are regulated by the same transcriptional pathways associated with biotic and abiotic stress, notably those using the WRKY and TGA transacting factors.^43^ Such observations help to explain the enhanced expression of enzymes such as glutathione transferases by both pathogens and safeners, although the differential induction of isoenzyme types in each case demonstrates their specific regulation.^44^ Intriguingly, the potential for plant–microbe signalling to contribute to herbicide tolerance is a two‐way process, extending to the host potentially manipulating the endophyte population to enhance protection. For example, plants exposed to pathogen stress actively excrete chemicals such as malic acid that increase biofilm formation by rhizobacterial species (e.g. *B. subtilis* FB17), which stimulates the plant's endogenous defence responses as well as directly metabolising herbicides.^45^

In spite of the associative evidence that endophytes can directly contribute to herbicide tolerance/resistance in host plants, there are only a few examples where this has been reported in the field. In one classic example, infection with the endophytic fungus *Neotyphodium occultans* was shown to correlate with herbicide resistance in populations of annual ryegrass (*Lolium rigidum*) in Australia.^46^ Originally identified as a fungal endophyte in wild populations of *L. rigidum*, controlled infection studies demonstrated that the introduction of *Neophytodium* spp. into the related wild grass *L. multiflorum* enhanced stress tolerance and growth.^47^, ^48^ In *L. rigidum*, infection with *Neophytodium* spp. resulted in an increased tolerance to the graminicide diclofop‐methyl in populations that were normally susceptible to the herbicide.^46^ However, this effect was not observed in *L. multiflorum* populations that were already naturally tolerant to diclofop‐methyl. These very interesting observations linking *Neophytodium* species with herbicide resistance in *Lolium* have not received the further attention they deserve. Many wild grasses contain microbial endophytes, and their potential to contribute to the steady rise in non‐target‐site herbicide resistance in these weeds certainly warrants investigation given the global scale of the problem. A closer examination of the literature may suggest a mechanistic link between fungal endophyte infection and herbicide resistance in wild grasses. Grasses infected with fungal endophytes show enhanced growth and seed production as compared with uninfected plants, which has been ascribed to the reactive oxygen species (ROS) scavenging activity of the symbiont.^49^ Importantly, one of the traits associated with non‐target‐site‐based herbicide resistance in wild grasses is linked to enhanced antioxidant content and ROS scavenging capacity.^50^ Such cytoprotective responses appear to be important in protecting plants from the downstream toxic events triggered by herbicide action.^51^ Thus, ROS are frequently generated as a secondary toxic consequence of the derailment of photosynthesis and primary metabolism owing to a breakdown in membrane integrity and associated redox processes. While such an association between endophyte‐endowed enhanced ROS defence and herbicide resistance remains to be confirmed, grass species harbouring *N. occultans* are endowed with increased defences to biotic and abiotic stress that can even contribute to host dominance over other plant species.^52^, ^53^ As an alternative to actively priming host defences, direct gene transfer should also be considered to be a route by which endophytes can contribute to herbicide tolerance in the plant host. For example, the transgenic introduction of the anti‐apoptotic baculovirus *p35* gene into passion fruit plant resulted in enhanced tolerance to glufosinate.^54^ Intriguingly, this suggests that plant viruses containing anti‐apoptotic genes that facilitate infection could also protect the host against herbicide‐induced cell death. Endophytes also have the potential to influence the chemical control of weeds by having a regulatory role in controlling seed dormancy. A recent study demonstrated that *L. rigidum* seed bacterial populations contribute to dormancy, with infected weeds proving more difficult to control over time owing to their persistence in the soil.^55^

## 5 THE EVOLUTION AND FUTURE APPLICATIONS OF PLANT–ENDOPHYTE‐BASED CHEMICAL TOLERANCE

The appearance of synthetic xenobiotics in plant–microbe environments is a recent phenomenon on an evolutionary timescale. Therefore, the capability of both free‐living and endophytic microbes to degrade herbicides is likely to be a modern adaptation of pre‐existing natural xenobiotic/stress tolerance inducing pathways. In the natural world, plants and soil microbes are constantly exposed to natural toxic allelochemicals, such as benzoxazinoids, phenolics and glucosinolates, that are exuded by competing plant species and by some free‐living and symbiotic bacteria.^56^ Such microbial traits can be exploited by plants that attract rhizobacteria, aiding them in plant–plant competition.^35^ Based on the intensive use of pesticides and herbicides in agriculture in the last 50 years, it is clear that we are now introducing new selection pressures not just on weeds, pests and pathogens but on potentially beneficial microbial symbionts and endophytes.

This review has highlighted some of the potential ways in which microbes can directly influence crop and weed responses to herbicides, and this would seem to be a fertile area for future research. Attention should be drawn to cases where unsuitable endophytic organisms are susceptible to and can increase herbicide toxicity to host plants, as in the case of mycorrhiza‐infected apple trees, possibly owing to increased herbicide uptake.^57^ Based on their significant impact on crop growth and health, it is not surprising that beneficial endophytes are already being developed for new applications in crop improvement, with a focus on screening for new strains and methods of selective inoculation.^3^ Protective traits enhanced or endowed by endophyte or rhizobacterial strains may be dependent on the host plant genotype, as shown previously.^36^, ^58^ Breeding crop varieties to optimise beneficial interactions from microbes would be an expensive but worthwhile goal. Perhaps the greatest benefit of using endophytes to manipulate herbicide tolerance in crops lies in using genetically modified microbes that can be produced at a fraction of the cost of generating GM crops, which is seen as a potential stumbling block for their usage by growers.^59^ The review has already identified useful traits in natural endophytes, such as herbicide detoxification and the manipulation of protective host stress tolerance mechanisms, that could be further exploited in a wider range of microbes, effectively expanding the range of crop species amenable to the technology. There are also a large number of detoxification mechanisms available in free‐living microbes used in phytoremediation that could be exploited for metabolising chemistries that are otherwise resistant to plant based biotransformation pathways, such as those acting on nitrile and halogen functions or catalysing aromatic ring fission. These detoxification mechanisms can be further optimised using mutagenesis or forced molecular evolution to increase their efficiency or specificity. From a regulatory and safety perspective, the use of such GM endophytes would require a robust technology to prevent escape into the environment. We have termed this obligate linking of the endophyte to the crop ‘biotethering’ and propose a synthetic biology approach to engineer such a trait in the microbe. Plant symbionts and pathogens already utilise a range of natural products to locate and infect host plants. Classic examples include the isoflavonoids produced by legumes to attract nodulating bacteria and the oxidised phenolics that stimulate *Agrobacterium* infection. Using a synthetic biology approach, we would propose to engineer a chemical sensing pathway, specific to the recognition of natural products derived from the host crop, which would then be linked to controlling the expression of a gene involved in an essential metabolic pathway. In the absence of the host natural product, the microbe would be unable to survive, thereby ‘biotethering’ the endophyte to its host. Such an approach circumvents the need for genetic modification of the host plant, effectively allowing fine tuning of the specificity of plant–microbe interaction while avoiding the potential risk of modified plant gene introgression into other varieties or weeds that was addressed in depth by Gressel.^60^ Previous attempts at crop plant inoculation with modified endophytes have included the pressure infiltration of endophytes into seeds.^61^ Potentially, rhizobacterial inoculum genetically engineered with biotethering technology could be incorporated into a seed coat for infection upon germination, although this concept has yet to be tested for selectively endowing host plants with herbicide metabolic and/or tolerance traits.

While the regulatory framework and public acceptance of such approaches are yet to be tested, the associated technology is already well developed and would be considerably cheaper than developing new crop traits either through marker‐assisted breeding or transgenesis. Intriguingly, such herbicide selectivity traits may already have been selected for in plant‐associated microbes in the course of normal crop protection. However, these microbial symbionts and the protective traits they impart remain undiscovered in the absence of major screening programmes. In the absence of new herbicide chemistries and modes of action, such approaches are likely to become increasingly attractive in working with an ever decreasing range of registered products and having to deal with the steady growth of resistance in weeds.
